# A Treatise on the Diseases and Special Hygiene of Females

**Published:** 1846-04

**Authors:** 


A Treatise on the Diseases and Special Hygiene of Females. By *Colombat de l'Isère.* Translated from the French with Additions, by *Charles D. Meigs*, M.D., with Woodcut Illustrations. Philadelphia: Lea and Blanchard, 1845.

A Practical Treatise on Inflammation, Ulceration, and Induration of the Neck of the Uterus, with Remarks on the Value of Leucorrhœa and Prolapsus Uteri as Symptoms of Uterine Disease. By *James Henry Bennet*, M.D. London: Churchill, 1845.

We learn something of M. Colombat from the dedicatory letter of the translator of his work. Dr. Meigs indeed has forestalled the reviewers, for not only does he speak of M. Colombat's "enormous toil" and his "elaborate and judicious collation of authorities"—a thousand of whom are indexed up like an army-list at the end of the book—but he tells us that he has himself contributed 100 pages of new matter to M. Colombat's 600, and he adds an opinion which bears the genuine feature and expression of the fag-end passage of a favourable review, viz. that, "as a text and table-book for the student and practitioner, Dr. M. does not think it has an equal in its department." We must at the outset take the liberty to demur to Dr. Meigs' judgment in this respect. To us it appears that some of the enormous toil might well have been expended in pruning the work, and that the collation of authorities would have been more judicious had they been reduced in numbers and used with more point and illustration.

Dr. Bennet's work is a monograph on inflammation of the cervix uteri, and we think he has very ably supplied a deficiency in this part of medicine. The subject is very important—it embraces simple inflammation of the uterine neck, and inflammation with its sequelæ, induration and ulceration, and some specific forms of ulcers from syphilis and cancer. We do not hesitate to say that Dr. Bennet has treated these subjects more clearly and faithfully than any author we are acquainted with, and we have read his treatise with much real satisfaction.

It appears that Dr. Bennet has gained his knowledge on uterine pathology principally from the Parisian hospitals, and that, in his official capacity as an *interne*, he has not only enjoyed an extensive and varied field for observation, but has been in the habit of analysing the cases for the instruction of his clinical pupils. We regard this plan as an admirable discipline for an author, and we find the benefit of it in the lucid and faithful descriptions which are the pleasing characteristics of Dr. B.'s book. It is a luxury we covet to meet with a clinical treatise, a plain transcript of what may be verified almost daily at the bed-side—well illustrated but not overdone with cases, and with a due complement of authorities, if they can be made of service. But to fill out a book with a large gathering of cases, and to parade authorities by the squadron is, in the one instance, to be guilty of useless repetitions, and, in the other, of a display of false learning, So far as imparting information goes, they are both equally inoperative. We think that although there is much that is meritorious in M. Colombat's work, yet that it has a vice within it which is snfficiently indicated in the introduction, when M. Colombat tells us that the fourth section contains "*nearly two hundred pages.*" It is this effort to multiply pages, to expand instead of condense, which makes the book wearisome to read, and detracts very much from its usefulness. We feel that Dr. Meigs has accomplished a somewhat tedious task in translating and editing the work; and, judging from the general character of his additions, we are almost tempted to wish that he had himself written an independent treatise, and have suited his own taste and convenience about giving M. Colombat an English dress. If the object of Dr. Meigs was to render the book available for the student and practitioner, we fear, as it at present stands, that it will draw too largely on the time and patience of both.

We may just notice that both Dr. Meigs' and Dr. Bennet's English is somewhat peculiar; the former, for want of a better title, we must call American, and the latter, has yet to be gleaned of a few French characters which hang about it. With this very occasional exception Dr. Bennet writes clearly and well, and we are very willing to put up with a little foreign interpolation, when we remember that the matter of the essay was derived from France, and is much improved for having been strained through an English mind.

M. Colombat lays out his treatise on a large scale, and in his first Chapter he considers the Phenomena of Menstruation and Gestation, and the Cessation of the Menses. These subjects are imperfectly described, and Dr. Meigs supplies some of the most important omissions. The influence of climate on menstruation and the reputed cause of the eruption and recurrence of the menses from the ovarian ponte, have been left for Dr. Meigs to discuss. In answer to inquiries from Dr. Meigs, Dr. Vargas of Caraccas in Venezuela, states that in that city menstruation commenced in 70 per cent. of women from 13 to 15 years of age, in 10 per cent. from 11 to 12, in 20 per cent. from 16 to 18, and in very rare cases from 19 to 20 or even 21. The most common critical age was from 45 to 48, with rare cases varying a few years before or after this time.

In the woodcut, which gives a side view of the female pelvis, we notice an anatomical error which is seen in several other books. The posterior lip of the uterus is made longer and more projecting than the anterior. This ought to be reversed.

M. Colombat gives a Chapter on the best mode of examining the female generative organs by the touch and by the speculum. He speaks favourably of the standing posture, with the body bent forward to relax the muscles, for ascertaining the size, weight, direction and elevation of the womb. There can be no doubt that the influence of gravity which is obtained in this position is of great use in the physical examination of the womb, and we are much in the habit of practising it. The horizontal posture, which is too exclusively adopted in this country, frequently effaces for a time some of the diseases, which are felt only in the standing posture. Thus, partial prolapse of the uterus is relieved by lying down, and some of the moveable tumours are displaced beyond the reach of the examining finger under the horizontal posture. The effect of the vertical position may be aided by a voluntary effort at bearing-down, which often serves to bring the lower part of the body of the womb well within the influence of the finger, which can thus more readily appreciate its weight and direction. The co-operation of the two hands by pressure over the pubes, which is effected easily in this position, contributes to the accuracy of our diagnosis.

M. Colombat finds out that the invention of the speculum is of the highest antiquity, and that an inhabitant of Syria who settled in Rome in the reign of Domitian first made it known. This is a most happy circumstance, for it has saved us, we hope, from one of those scrambles after notoriety which make claims to priority in an invention or observation, the too frequent subjects of fretful and acrimonious controversy. We fear only with reference to the speculum, that, like the long forceps, it may be endlessly varied, not with any hope of improving a very simple instrument, but to gratify the little vanity and very moderate ambition of new-formed aspiring professors. M. Colombat figures a speculum with eight blades, which we have never seen; and as a pleasing contrast we have the simple conoidal tube of metal known as Recamier's speculum—which, in M. Colombat's words, "to a great simplicity of construction it joins the advantage of being easy of application, of shewing better than other instruments the cervix, by reflecting the light upon it. Besides, as it is whole, the mucous membrane of the vagina cannot mask the os uteri by intruding itself between the open branches of speculums composed of several pieces."

These are most desirable advantages, and, in our own experience, the common cylindrical speculum, either of metal, or, as we prefer it, of glass, is the most simple, useful, and easily-applied instrument that we know of. It is quite sufficient for the examination of the cervix and vagina in most of the diseases of these structures, and for the application of local remedies for their relief. Sometimes we find the metallic two or three bladed speculum of use, when we want to expand the upper part of the vagina, so as to get room to twist off a small polypus for instance, or to get at a vesico-vaginal fistula, &c. One of the practical difficulties which is met with in the exploration with the speculum, is to get the cervix nicely within its cylinder, and, for correcting any difficulty in this respect, M. Colombat has invented a kind of concave lever, with which he can draw this part within the speculum. We have seen other contrivances for this purpose, such as a long hole in one part of a metallic tube, or a moveable shutter, which may be drawn out of the speculum, and thus allow a finger to enter and reach and redress the cervix. We usually find that a little patience, with a previous arrangement of position, according to the circumstances of the case, will generally be sufficient to effect this purpose, with the ordinary cylindrical glass speculum without these mechanical complications. With reference to the position of the patient, either the back or side, or in some cases on the elbows and knees, on an ordinary bed somewhat raised, have each their facilities for exploration, and ought to be selected according to the peculiarities of the case. We have frequently adopted with advantage the half-sitting, half-reclined posture, with the pelvis brought to the extreme edge of a chair, and the trunk thrown backwards, especially when we have had to perform the very useful little operation of scarifying the cervix, which may in this way be completed without any mess or trouble. Before quitting this subject, we have a word to say upon the reputed indelicacy of the use of the speculum. "I have often been told," says Dr. Bennet, "that females in this country will not submit to treatment when afflicted with uterine disease." We have been told so too; but, like Dr. Bennet, we have not found it. We have heard this accusation of indelicacy from practitioners whose diagnosis of uterine disease has, through perhaps a long life, been sought exclusively by the sense of touch, and who declare that, for all *practical* purposes, this is sufficient. We have heard it—and seen it written too—by persons who are evidently quite ignorant of the difficulties and responsibilities which attend the practice of this department of medicine. We well remember a like accusation of comparative inutility being preferred against the stethoscope by a venerable hospital physician, whom we have seen walking around the wards with a bunch of flowers stuck conspicuously in the cylinder of the stethoscope, and, when appealed to with reference to its alleged value in the diagnosis of chest-diseases, used to say emphatically, that there was "more sound than sense" in it. Like our veteran accoucheur, he regarded his ordinary means of diagnosis quite enough for all practical purposes, without the education of another sense to assist him. Now we, who are of a medium age, find that we cannot so readily dispense with one of our senses. If the uterus or its contents emit a sound, we like to hear it—if it is within reach, or by any available means can be brought within reach, we like to feel it—and if, again, by direct or mechanical means, we can bring any part of it within our view, we like to see it. And we are quite sure that we can now cure diseases of the womb which were not within the compass of our resources fifteen or twenty years since, and that the advantage to our patients is quite commensurate with our acquired facilities in physical diagnosis. To impute indecorum or indelicacy to the use of the speculum, is, in fact, to raise a false alarm, and with just about as much sense and propriety as it would be to reflect upon a man for examining the rectum both by sight as well as by touch, when a fistula is sought for. The very suggestion of the thing is by far the most indelicate part of it—and the mind in which such a suggestion could be raised or lodged, has just that kind of taint which ought practically to disqualify it for the duties which belong to an obstetric practitioner.

The plan and contents of M. Colombat's work are thus announced.

"In order to facilitate the study of the lesions of the genitalia, and to group them, as far as practicable, according to the natural order they ought to occupy in a general system of pathology, of which they constitute but a trifling portion, we have made a classification in which we divide them into sections:—as, 1, *Lesions of form and development.* 2. *Lesions of situation.* 3. *Physical lesions.* 4. *Vital lesions.* 5. *Lesions of functions.* 6. *Lesions appertaining to reproduction.*

"Although we might be disposed to look upon this classification of female diseases as more rational than those proposed by our predecessors, we are far from deeming it perfect and unattackable. But we are somewhat reassured, in regard to its imperfections, by the consideration that there is no perfect classification in pathology; and further, that all writers on female disorders have, like ourselves, met with some shoals which it is impossible to avoid.

"*Synopsis of the Diseases of Females.*

| | |
|---|---|
| I. Section. *Lesions of Form and Development.* | "*Comprising all cases of vicious conformation, whether congenital or accidental, of the sexual organs and their appendages*—among which we enumerate absence of the labia, cohesion of the labia, excessive magnitude of the nymphæ, cohesion of the nymphæ, excessive development of the clitoris, imperforation and stricture of the urethra, absence of the vagina, contraction of the vagina, narrowness of the vagina, obliteration of the vagina, imperforation of the vagina, obturation of the vagina, congenital opening of the vagina into the rectum or bladder, absence of the womb, bifid womb, incomplete development of the womb, congenital occlusion of its neck and its accidental obliteration, and, lastly, imperforation of the Fallopian tubes. |
| II. Section. *Lesions of Situation.* | "*Comprising all cases of displacement and deviation of the genito-urinary organs of the female*—among which we arrange hysteroptosis or prolapsus of the womb; anteversion, retroversion, anteflexion, retroflexion, inversion, obliquity, elevation and immobility of this organ; hysterocele and all the hernias of the womb and ovaries; vaginal cystocele and enterocele; vulvar enterocele and cystocele; prolapsus of the urethral mucous membrane; prolapsus of the lining membrane of the vagina, and invagination of the canal. |
| III. Section. *Physical Lesions.* | "*Comprising all cases of lesion of continuity, and the accidental introduction of foreign bodies*—among which are found wounds, contusions and lacerations of the vulva, the fourchette, the vagina, the uterus, and the rupture of the womb; vesico-vaginal, urethro-vaginal and recto-vaginal fistula; and, lastly, foreign bodies accidentally introduced into the genital cavities. |
| IV. Section. *Vital Lesions.* | "*Comprising the phlegmasia, the transformations, and all the pathological products and degenerations of texture,* such as phlegmon, carcinoma, œdema, cysts, varix, fibrous and sanguine tumours of the labia. Inflammation and fungus of the nymphæ, carcinoma of the clitoris and meatus urinarius, erysipelas, prurigo, venereal chancres and syphilitic excrescences of the vulva, acute vaginitis, chronic vaginitis, and all the white discharges; acute and chronic metritis, uterine phlebitis, ulceration, excoriation, fungous tumours and engorgement of the cervix uteri; scirrhus, cancer, carcinoma, putrescence, softening, dropsy and tympanitis of the womb; metrorrhagia, polypus, fibrous tumours, calculus, hydatids, sanguine and lymphatic concretions formed in the cavity or in the substance of the womb; scirrhus, cancer, encysted tumours, and dropsy of the ovary; and, lastly, cancer of the breast. |
| V. Section. *Lesions of Functions.* | "*Comprising the neuroses, neuralgia, and functional derangement of the female organs of generation,* such as cessation of the menses and all the sympathetic phenomena of the change of life. Hysteria, nymphomania, false pregnancy, hysteralgia, anaphrodisia, inertia of the womb, mastodynia, chlorosis, dysmenia, amenia, amenorexia, amenorrhœa, dysmenorrhœa, menorrhagia, menostasis, and all the anomalies of menstruation. |

**VI. Section.**

*Lesions appertaining to Reproduction.*

"*Comprising the accidents and all the sympathetic phenomena of conception, pregnancy, labour and lactation,* among which are false germs of moles, extra-uterine pregnancy, abortion, strange appetite, anorexia, odontalgia, ptyalism, convulsions, vomiting, nervous cramps of the stomach, nervous colic, constipation, diarrhœa, tenesmus, dysuria, ischuria; the hernias which sometimes complicate pregnancy; dyspepsia, cough, hæmoptysis, hæmatemesis, epistaxis, sanguine plethora, palpitations, syncope, varices, hæmorrhoids, œdema of the limbs, cephalalgia, vigils; neuroses of sight, hearing and smell; neuralgia of the loins, kidneys, groins, pubis, labia and thighs; contusions and lacerations of the genital parts; inversion of the womb and vagina, puerperal peritonitis, milk fever, phlebitis of the uterine and ovaric veins, of the inferior cava and the crural veins; neuritis of the sciatic, crural and sub-pubal nerves; painful œdema, phlegmonous abscess of the mons and labia, of the psoas and iliacus muscles; absence, diminution, suppression or excess of the lochia; miliary eruption, polygalactia, agalactia, retention of the milk in the breast, involuntary flow of milk, alterations of the milk; and lastly, mammary abscess, mammary fistula, cracks, excoriations, flattening, imperforation, absence and multiplicity of the nipple." P. 74.

In the first section, amongst the lesions of form, M. Colombat describes imperforation and stricture of the female urethra, and the best modes of relieving it—noticing, also, the natural or supplemental relief, which an opening of the urachus at the umbilicus occasionally offers. The disease is regarded as the result of congenital deficiency, and the acquired stricture in the adult urethra is not mentioned. An imperforate stricture in a grown-up woman we have never met with; but impediments to the passage of urine, from thickening and contraction of the canal, is a kind of stricture which might well be described. The primary effect of this is simple dysuria—but the straining efforts to relieve the bladder greatly aggravate the affection. The inferior part of the bladder becomes prolapsed, and sometimes the contiguous portion of the urethra—one or both are forced out through the vagina—the urethra gets doubled on itself, curving its canal—the structures become œdematous—straining efforts increase—an involuntary dribbling of urine sometimes comes on—and the bladder is only partly emptied—and that too with much difficulty. The secondary effects of this form of disease are the most troublesome, resulting principally from the displacement of the parts, and they cause much local and constitutional suffering.

This section contains a full chapter on imperforate and contracted vagina, with the treatment adapted for its relief. M. Colombat figures some instruments for the division of the adherent walls, which he invented for the purpose.

The first lesion of situation which is described in the second section is the falling of the womb. We had always thought that prolapsus uteri, as a name for this affection, contained quite enough of Latin and learning to satisfy the most exacting taste for this kind of nomenclature, but M. Colombat has taken the trouble to build up a fresh title quite eclipsing the former one, which is no less a puzzle for the student than Hysteroptosis.

"Hysteroptosis or falling of the womb, is the displacement of the viscus downwards, and it may be complete or incomplete.

"In the former case the organ escapes entirely from the pelvis, and may be seen completely outside the vulva—in the latter case, on the contrary, it projects more or less considerably into the vagina, but does not descend below the inferior strait. Prolapsion of the womb may take place not only during pregnancy and after delivery, but also while the womb is non-gravid, for it has been met with even in virgins."

We gather from M. Colombat that a prolapsus of the womb in a virgin is a far less frequent disease in France than it is here. He says that Mauriceau, Saviard, Monro, and De Graaf, are the only authors who have reported cases of complete prolapsion of the womb in the virgin state. That this displacement occurs far more rarely in single than in married women is sufficiently obvious; but we have—we may say—*frequently* seen it in the former, and we have now three cases under care of the complete descent of the womb in young unmarried women. M. Colombat justly enough regards, as occasional causes of the affection, all movements which require sudden, frequent, and powerful contractions of the diaphragm and abdominal muscles. And it is the constant operation of this class of causes in the hard-working women of this metropolis which bring to our hospitals cases of complete prolapse in virgins. With Lisfranc, and, we may add, Dr. Bennet, our author looks upon uterine engorgement as the principal cause of the displacement in question. But, in the cases we are speaking of, there is no previous congestion present—it is the sole result of the muscular effort. One woman is moving a heavy paralytic patient, and the womb falls—another is putting up the curtains on a bed, and the womb comes down—another, while carrying a basket on her head, or a heavy tray in her hands, stumbles, and with difficulty recovers herself, and this form of hernia takes place. In neither case will the womb have been too heavy, or the vagina unduly lax from marriage or child-bearing, or the floor of the pelvis weak; but all these natural impediments to the accident be overcome by the powerful contraction of the abdominal muscles.

Like most other writers on the subject, M. Colombat describes three degrees of prolapse—and the character of the symptoms of the second degree, with the difference in them when the prolapse has been caused suddenly, and when it comes on gradually, are thus noticed.

"Most of the symptoms of prolapsus in the second stage are produced by the pressure of the womb upon the surrounding parts, especially the bladder and rectum, or by the stretching of the uterine ligaments. This is put beyond question by the fact that all the symptoms are diminished by rest, and particularly by rest in the horizontal posture, whereas their violence is redoubled by standing and walking. Where the displacement has been gradually produced, the symptoms attending it are less severe than where it takes place suddenly; where it is suddenly produced, it is frequently accompanied with long, protracted faintings, violent floodings, severe pain in the pelvis, vomiting, and sometimes even an intense attack of peritonitis. But, on the contrary, where the displacement takes place slowly, these phenomena are rarely observed, because the organs, having slowly abandoned their natural situation, become, in a measure, accustomed to the unnatural situation they have assumed." P. 123.

With the object of elucidating the mechanism of the displacements, Dr.

Meigs describes the natural supports of the womb. He regards the ligaments as so many stays or restraints upon the womb—the broad ligaments preventing its lateral inclination, and the round ligaments opposing its falling backwards when the bladder is too full ; while its vertical position is maintained by the structures forming the septa before and behind it. We cannot forbear to copy the following sensible observations, in which we perfectly accord.

"It has long since seemed to me," says Dr. M. "that the writers upon prolapsion have lost sight of one most important element in the pathology of the case, and that is, the state of the muscles within the pelvis. They ought to have observed and remarked upon this important physiological law, viz. that the diaphragm has an antagonist force in the floor of the pelvis, which is partly muscular, and that where that muscular floor retains, together with the other tissues of the perineum, its full power and energy, there can be no prolapsion of the womb. The repeated distensions and dilatations to which the floor of the pelvis is subjected in labour, cannot but tend to debilitate it and overcome its power of resisting the antagonization of the diaphragm and abdominal muscles, and as the parts that sustain the womb depend for their own support on the firmness of the tissues composing the pelvic floor, it is clear that the womb may go down with them and it. But let it be observed that the pelvic floor itself is dependent, not merely upon its textural contractility, but on the muscular power of the levator ani muscles—muscles which relax in labour, and in defecation, by a spontaneous power of relaxation, in order to admit of the descent and protrusion of the pelvic floor in question, and which is restored to its proper level, and drawn upwards, and kept there by the sole power of the levator muscles. Hence it is, I think, clear, both by reasoning and by observing clinically the facts in the case, that the weakness and loss of power of the levator muscles have much to do with the pathology of prolapsus uteri.

"It will not, I suppose, have escaped the observation of all persons of experience, that in bad cases of prolapsus, the perineum is thin, feeble in its tension, and that the whole perineum is at a lower level than in those who do not labour under prolapsus.

"It is essential to remark that weakness and prolapsion of the womb are apt to follow bad labours, and to be coincident with the signs of general debility of the muscular apparatus of the whole body. It is not, indeed, met with, except very rarely, in the strong, active muscular subject, and that even in these subjects the prolapsion is rather a state of immobility or fixedness of the womb at a low level, than a real prolapsus." P. 124.

The symptoms which accompany prolapsus in its several degrees are fairly described, although we are much surprised at some obvious omissions. The tumour of the completely procedent womb is stated to be strangulated, oval or globose, but most commonly conoidal; but the direction of it downwards and backwards, in a curved form, so that the os and cervix are best seen when the woman lies on her side and the tumour is examined from behind, is not mentioned. The orifice of the cervix is said in these cases to be "always much contracted." Our own experience is rather the reverse of this. We have noticed the os more gaping than usual, and certainly not smaller than in its natural position. The changes on the inverted surface of the vagina, from being wetted with urine and rubbed by the thighs, are described, but we do not see any special mention of the ulceration which occurs so very frequently at the most depending part of tumour. It is impossible for any one who has been in the habit of seeing uterine disease at all extensively, not to have met with numerous instances of this ulceration, and we do not understand how it could have escaped M. Colombat's observation.

In speaking of prolapsus in pregnant women, M. Colombat says that—"when the pregnancy has reached its full term, the escape of the fœtus may be facilitated by gradually dilating the os uteri, and the placenta should be removed by the introduction of the hand into the womb, so as to take it, and not by pulling at the cord." Dr. Meigs has very properly corrected the last error of practice, and we think the artificial dilatation may well be dispensed with. M. Colombat appears to be quite unaware of the effect of labor pains in correcting this hernia. A perfect prolapse of the gravid womb will, under the action of uterine contraction, be replaced, the womb ascending at each pain. We do not mean to say that this auspicious result is invariable, but we have known instances of it, and it affords a strong argument against artificial attempts to open up the passages.

We must refer our readers to a full and valuable chapter on vesico-vaginal and recto-vaginal fistulas. These diseases have afforded M. Colombat full scope for the indulgence of his taste for contriving instruments. Dr. Meigs relates a very interesting case of recto-vaginal fistula, the result of an abscess, which was successfully treated by Dr. J. Rhea Barton of Philadelphia.

M. Colombat makes no mention of rheumatism of the gravid womb, but the deficiency is supplied by Dr. Meigs, who has translated Cazeau's article on this affection, which is the most descriptive and complete that we know of. We feel that any attempt to analyse or condense this article would be unsuccessful, and although the quotation of it is long, we are assured that our readers will be gratified by perusing it.

"*Causes.*—All such circumstances as are favourable to the development of rheumatic affections, may likewise lead to an attack of rheumatism of the womb. Thus exposure, whether momentary or prolonged, to dampness and cold, insufficient clothing, sudden transposition from an elevated to a very low temperature, and all other causes, constitutional and atmospheric, regarded by medical authors as occasional or predisposing causes of rheumatism, may also produce that of the uterus. But, besides these general causes, there is one peculiar to the malady under consideration. I allude to the facility with which this organ, under the thinned integuments of the abdomen, feels the impression of cold in the latter months of pregnancy; the abdomen being guarded, when it encloses the uterus by extremely light garments, which are closely in contact with it, and the antero-sacral region being often badly protected by jackets of insufficient length.

"*Symptoms.*—Rheumatism of the womb often attacks persons constitutionally predisposed to nephritis. It may coexist with a general affection of the same nature; but, in a majority of cases, the uterus alone, and the adjacent structures, are the seats of the disorder. It has, besides, been frequently found to be a consequence of the sudden cessation of rheumatic pain, originally situated in some other part, and suddenly transposed to the womb. Whatever may be the mode of its onset, the disorder is easily recognised by very decided characteristic features. Its principal symptom is pain, where not the least violence has been offered to the organ, the womb becomes the seat of a general or partial pain, the intensity of which varies from the very slightest sense of weight up to the most insupportable agony. It may affect the uterus wholly, or only attack some particular part of it, as the orifice, the fundus, or the cervix. Where the rheumatism is fixed in the fundus only, the pain is felt in the region above the umbilicus. It is increased by pressure, by the contraction of the abdominal muscles, and sometimes by the mere weight of the clothes; the patient, often, is unable to move; if the disorder is seated lower down, there are shooting pains that run from the loins towards the pelvis, the thighs, the external genitals, and the sacral region, along the ligaments of the uterus. Lastly, when the cervix is the affected part, it may be known by the vaginal touch which gives rise to excessive suffering. But of all the causes that serve to exasperate the pain, none is so distressing as the incessant motions of the child.

"Like other rheumatic pains, those of the womb are moveable, and are observed occasionally to pass suddenly from one portion of the organ to another. They often suddenly cease, and proceed to attack some other organ. This is most apt to happen, where the uterine rheumatism has been preceded by a fixed pain of some other part of the body, and where remedies are in use calculated to recall the pain to its original seat.

"These pains are characterised by frequent exacerbations that are variable as to their duration and intensity; according to the stage of the malady, they are succeeded by remissions, during which the patient scarcely complains of a vague sense of weight.

"The pains of uterine rheumatism are generally attended with a degree of recto-vesical tenesmus, which is violent in proportion to the severity of the pains and the approximation of the seat of the rheumatism to the lower segment of the organ. In such cases the patient is tormented by perpetual desire to urinate. The discharge of the urine is accompanied with smarting pain, sometimes with severe pains, and in some instances the discharge cannot be effected at all; the efforts to discharge the contents of the rectum are, in some cases, equally fruitless. Most of the German authors attribute this double recto-vaginal tenesmus to the rheumatic disease, which is not always confined strictly to the uterus alone, but may likewise invade the circumjacent organs. M. Stolz seems disposed to think that it arises from the close sympathetic relations of parts so nearly approximated to each other. Should these new pains be owing to a vesical or rectal rheumatism, those of the womb would disappear, or at least be diminished in degree, according to the views of M. Salathe in his Thesis.

"It is to be supposed that there is a degree of heat and swelling of the affected parts; but it is easy to perceive the difficulty of absolutely determining this point, one which we are compelled to admit from analogy.

"Pains of such violence, situated in an organ so important, must of necessity produce a pretty severe general reaction. The disorder, like most of the inflammatory diseases, generally commences with a slight rigor, which lasts fifteen or twenty minutes. The succeeding fever diminishes, or may even wholly cease during the interval between the attacks, yet while they last it is commonly quite severe; the pulse is hard and frequent, the face flushed and excited, the tongue red and dry, the thirst urgent; the skin is hot, and the patient is often found to be extremely agitated and restless. Towards the close of the paroxysm, there frequently supervenes a copious sweat, which seems to be the harbinger of a decided improvement. After this, these general symptoms are appeased, together with the uterine pains, only to re-appear with them, after the lapse of a few hours, or even of several days.

"1st. *Influence of Rheumatism on the progress of Pregnancy.*—Where the attacks may have persisted for a length of time, or where they have been very violent, they are followed by uterine contractions, and may, in this way, bring on premature delivery. In such a case the patient suffers from severe tensive pain. This feeling of tension is not equable, for it rises to a great height, and then subsides—to begin again and pursue the same course at different intervals. At first the womb becomes partially, and afterwards universally hardened during the pain. The cervix becomes rigid and partially dilated, but its dilatation is at first slow and difficult, and its subsequent progress does not correspond with the pace of the pains. The abortion, with which she is now menaced, is more likely to take place in the febrile than in the apyretic form of rheumatism. Indeed, abortion is not so common an occurrence in the case as might be presumed. In some instances the os uteri has been observed to dilate to the extent of two or three centimeters in diameter, the bag of waters has been formed, and afterwards withdrawn little by little, the orifice closing again, and all symptoms of labour wholly to disappear. As long as the diameter of the os uteri does not reach the extent of five centimeters, we may reasonably hope to put off the labour. These uterine rheumatic pains may simulate labour pains, and lead to the belief that they are really labour pains, while in fact they are not at all so. The characteristic signs of the rheumatic pains, given in the following paragraph, should serve to prevent such a mistake. It is surely to mistakes of this kind, that we ought to refer those cases of supposed protracted pregnancy, and those instances of real labour, begun, and suspended again for weeks and even for months together." P. 289.

"2d. *Influence of Rheumatism upon Labour.*—An attack of uterine rheumatism generally retards the progress of a labour, and sometimes even renders the spontaneous expulsion of the fœtus wholly impossible. In addition to the general phenomena I have described, there are here some special ones to be met with. 1st. It is well known that a normal contraction does not begin to be painful until it has accomplished the greater part of its task, and is in the act of dilating and distending the os uteri: in other words, the true pains of labour do not begin until the instant at which the energy of the corpus uteri begins to overcome the resistance of the cervix. In rheumatism of the womb, on the other hand, the uterine contraction begins to be painful from the start, and before the least power is exerted on the neck; so that the cause of the pain is not in the violent distension of the orifice, but in the contraction itself, in other morbid circumstances, and in other relations of the nerves and contractile fibres of the womb. 2d. In a natural labour, the contractions commence at the fundus uteri, and are directed towards the lower segment. In rheumatism, instead of commencing at the fundus, they commence at the painful point, and run towards the neck in an irregular manner. Again, the pains exist before the contractions of the womb; and, under their influence, when they are established, acquire a high degree of intensity. Their violence sometimes arrests the contractions before they have run through their ordinary cycle. They are, in such a case, brisk, short, and grow less and less frequent. 3d. Towards the close of the labour, when the action of the womb requires to be sustained by the voluntary contraction of the abdominal muscles, the woman, for fear of increasing her sufferings, refrains from contracting her abdominal muscles, which causes the labour to be excessively slow. The patient is in a state of extreme anxiety; the frequent pulse, the hot skin, the thirst, the urinary tenesmus, are much augmented. When the sufferings are too much protracted, she at last falls into a collapse, (which is often a fortunate event for her;) during which the pain is suspended. Under these circumstances, a profuse sweat has been observed, which has had the happiest effect on the rest of the labour. But, in other instances, the womb grows more and more painful; it is rather in a state of permanent contraction, or fibrillar vibration, than of real contraction; the pulse becomes accelerated, and now the woman is under the influence of a metritis which renders the labour extremely painful.

"3d. *Influence of Rheumatism of the Womb on the puerperal functions.*—One may conceive, *à priori*, that uterine rheumatism, by causing irregular or partial contractions of the organ immediately subsequent to the birth of the child, might be the occasion of much difficulty in the delivery of the placenta; but this is not the place to discuss that point.

"In health, after the delivery, the womb contracts, and thus prevents hæmorrhage. But in rheumatism this return of the organ is very incomplete; it remains above the pubis, and is large. The after-pains are now very painful, and continue for a long time. The uterine vessels are less compressed, whence may arise very copious floodings. On the other hand, the state of suffering in which the organ is placed diminishes the lochial discharge, and, the secretion of milk. The persistence of abdominal pain, added to the symptoms of a general reaction, might lead to the diagnosis of a peritoneal inflammation, though none such should really exist.

"*Prognosis.*—Rheumatism of the womb is not a disease capable of causing the loss of the mother's life; but, from the pain it occasions, and the mistakes to which it leads, it nevertheless merits all the attention of the physician. In pregnancy, it may cause abortion; and though it does not generally exhibit itself until the sixth month, it is always unfortunate for the child to be born before full term. We have already remarked upon the unfavourable effect produced by the disorder on the course and character of labour-pains. On many occasions it has led to the necessity of artificial delivery. It may likewise render the delivery of the after-birth difficult, and derange the course of the phenomena that ought naturally to follow after the birth of the child. At this period it is often confounded with phenomena that are purely inflammatory, and is then treated by measures that are hurtful rather than beneficial.

"The disorder is for the most part less favourable when attacking at an early than at a late period of gestation; because it has a more unfavourable influence on the progress of the gestation as yet incompletely established and settled; and also because it has a tendency to be reproduced again and again before the completion of the term, and on account of its disposition to return during the labour, which it is apt to render laborious.

"*Treatment.*—1st. During pregnancy, blood-letting, intestinal revulsives (ipecac. castor oil), baths, opiated lotions for the abdomen—anodyne potions, sudorific drinks. Such are the measures which have been most constantly successful. In cases where the affection of the uterus had followed the sudden disappearance of a rheumatic pain of some other part, revulsives should be applied to the part first affected. 2d. During labour, the same means are applicable; should they fail, and the os uteri as to its dilatation admit of it, let the delivery be effected by means of turning or the forceps. 3d. After delivery, sudorific drinks, anointing the abdomen with opiated ointments, baths, leeches to the vulva, and when the lochial discharge has failed, ipecac. and opium combined." P. 293.

There is a very meagre and unsatisfactory chapter on redness, simple ulcerations, and eruptions upon the os tincæ, by M. Colombat, which Dr. Meigs has not extended or improved, and we are glad to be able to fall back upon Dr. Bennet's monograph for a full exposition of these diseases. In considering his subject, Dr. B. makes an important primary division into inflammation and ulceration of the cervix in women who have not conceived, and those in whom the uterus has performed or partly performed its function of gestation. The division is sound and practical. These diseases of the cervix are rare in the unimpregnated female—they are very common in women who have had families; in the former, the ulceration is superficial, limited to the mucous membrane—in the latter, the deeper tissues of the cervix become affected, and this part of the womb swells, hardens, and becomes massive and heavy. Secondary affections are produced by these diseases in both classes of women. Leucorrhœa is most commonly caused by them—and we perfectly coincide in Dr. Bennet's remark, that "a confirmed leucorrhœal discharge, whatever be its nature, is accompanied by inflammation of the neck of the uterus." We are constantly meeting with cases of long-standing leucorrhœa which have withstood the influence of astringents, &c. and which readily yield to treatment when that treatment is applied to the proper part—viz. the inflamed or ulcerated cervix. We do not hesitate to say, that what has been called by authors symptomatic leucorrhœa, is infinitely the most common form of leucorrhœa, and daily experience in the investigation of uterine disease convinces us that the simple benign ulcerations of the cervix are the most frequent structural lesions which occasion it. Prolapse of the uterus in its first degree is another of the secondary effects of the engorged and voluminous cervix.

Inflammation and ulceration of the cervix in women who have not conceived may be strictly local, or co-exist with general vaginitis, as in gonorrhœa. Leucorrhœa may be profuse or scanty; there is lumbar and deep-seated hypogastric pain, and sexual coitus is painful. Sometimes there is a vivid perception of heat at the upper part of the vagina. On examining the uterus by touch, its neck is felt to be hotter than the vagina; it has lost its unctuous feel; it is larger and less elastic. Dr. B. dwells upon the perception by the finger of a slight superficial induration just beneath the ulcerated point, as a correct, though not infallible, guide to the discovery of this form of ulceration, Pressure on the inflamed and ulcerated cervix will often, not always, occasion slight pain. The diagnosis from the finger is to be corrected and rendered more exact by the use of the speculum—when the upper or anterior lip of the cervix will be seen to be the most swollen, and when inflamed presenting a more or less red glistening hue. On its surface may frequently be seen small white or red vesicular or papular elevations—the result of the distension or hypertrophy of the mucous crypts. There are different appearances in the ulcerated surface varying with the stage and character of the ulceration. The os uteri is generally filled with a mass of transparent mucus. Menstruation is generally more painful than in a healthy state. Indeed, the occurrence of the various symptoms of painful and difficult menstruation, when coupled with a leucorrhœal discharge, may be considered in most cases as pathognomonic of inflammation and ulceration of the cervix.

The above is an abstract of Dr. Bennet's account of this disease in women who have not conceived—which he has illustrated with two well-selected cases.

When once the uterus has performed its great function of gestation—its increased vascularity, the softening and development of its proper tissue—with the accidents from child-birth and abortion expose the cervix to inflammation and its products. Dr. Bennet discusses somewhat at length the causes of ulceration after pregnancy. Various local irritants may excite it, as sexual intercourse, aphthæ, &c. and general metritis—occasioned by abortion or labor, localising itself under a chronic form in the cervix and giving rise first to hypertrophy and then to ulceration. While Dr. B. admits this last as a cause, he is disposed to think that the majority of cases begin with superficial ulcerations and abrasions, and that the hypertrophy and general induration of the tissues of the cervix follow as a consequence upon them. We quite agree with him in admitting both these states as causing the disease in question—but, so far as our own observation goes, we think that chronic metritis is the more frequent preliminary to the development of ulceration. Dr. B. supports his opinion by having noticed in recent cases that the extent and degree of the engorgement coincides with the extent and degree of ulceration—and he explains its frequent occurrence after labor or abortion, not as resulting from chronic metritis or morbid hypertrophy, but from physical injury which the cervix sustains from its rapid dilatation.

"Now it appears to me a necessary consequence of this rapid dilatation of a canal lined by a mucous membrane in a state of integrity, that it must inevitably in many—if not in all cases be accompanied by erosion, laceration and contusion of the membrane. In the majority of women, no doubt, these lesions disappear promptly. Cicatrisation taking place with the greatest ease, under the influence of the retraction of the tissues of the neck and of the reparative phlegmasia which sets up after delivery in the cervix, as well as in the body of the uterus. But if the physiological inflammation of the uterus which follows parturition should prolong its duration and assume a pathological character, if remnants of the placenta, or of the membranes left in the uterine cavity give rise, by their decomposition, to an irritating fœtid discharge, it is easy to understand that the lesions of the mucous membrane, instead of healing, will almost inevitably become the seat of inflammation and subsequent ulceration."

It is very obvious that all this is hypothetical, and is not based on observation. Dr. B. does not tell us that in the uterus of so many women who have died either during or shortly after delivery he has found these injuries, for if so, we should ascribe some weight to his argument. But our own experience on this matter is quite the contrary of this; and we do not believe that, as a normal consequence of the first stage of labor, the mucous membrane is lacerated, erosed or contused, or in fact sustains any notable lesion. "Physiological inflammation," "cicatrisation," and "reparative phlegmasia," we discard altogether as terms suitable to the description of the cervix after a natural labor. Nor do we gain much certain knowledge on this matter by Dr. B's observation that he has traced ulceration from within the fissure of the os to the surface of the cervix a few weeks after labor. It is more than probable that, in these and such like cases, the error arises from bad practice, such as frequent and rough examinations, pernicious attempts to dilate the os artificially, or the effect of Ergot of Rye when given during the first stage of labor. We have long been accustomed to regard these as fruitful causes of local ulceration and leucorrhœa, and we believe they quite account for the numerous cases which follow parturition. These tears and rents of the lining tissue of the cervix are not so likely to succeed abortion, and we are disposed to regard the ulceration in these cases as a sequel to a congested state of the cervix. There is not a week passes that we do not hear this kind of history from patients whom we see. One or more abortions, or *slight miscarriages* as they are delusively termed, have not been particularly heeded; and the women, being perhaps in indigent circumstances, have immediately resumed the hard and sustained fag of their daily duties. At first, the symptoms of a heavy and congested womb are either unheeded or patiently endured, then after a time follows leucorrhœa, sterility lasts for months, or even years; then the health suffers, and our advice is asked for the whites. Ulceration of the cervix with hypertrophy and induration are the real structural lesions. But the train of causation does not start with the ulceration, but we believe with congestion of the cervix, which is kept up and increased by walking about, &c., then the womb falls somewhat, the swollen cervix is constantly fretted and rubbed against the vagina, and ulceration is the result. We have at least been accustomed so to explain these phenomena, and we cannot as yet abandon our views to take up those of Dr. Bennet.

The symptoms which attend ulceration, &c. of the cervix in women who have borne children include those which have already been enumerated in the first class of cases. But in addition, we have symptoms arising from the mechanical action of a large heavy cervix, which causes the womb to fall down on the posterior wall of the vagina ; hence there is a sensation of weight and heaviness in the pelvis, increased lumbar, sacral and femoral pain ; sometimes the rectum is pressed upon, and at others the bladder is interfered with. By touch we feel how low the womb is, the lax moist state of the vagina, and the condition of the os. This part is found usually open, and the ulcerated surface imparts a soft, velvety, mossy sensation to the finger. Sometimes the os is gaping, at others, one or other, or both the lips are fissured and lobulated, the edge of the cervix everted, and the whole mass raised into soft granulations. By speculum, the ulcerated surface is exposed, and the various appearances which are seen in ulcers in other parts of the body, are fairly copied in the cervix of the womb.

Dr. Bennet has related several instructive cases, which mark well the prominent features of this form of ulceration, in its milder and severer characters. Before adverting to the treatment of ulceration, &c. of the cervix we wish to notice one or two observations which Dr. B. has made.

1. Sterility usually accompanies these affections.

2. Ulceration may attack the pregnant womb, and there is no need of alarm in the use of the speculum in these cases, or the application of suitable remedies.

3. A chronic hypertrophy may last after the cure of ulceration, and, if means are not adopted to get rid of it, ulceration will soon recur.

Our author's third Chapter is on syphilitical ulcerations of the cervix, which are very well described, but we must content ourselves with quoting the conclusions Dr. B. has arrived at.

1. The real classical chancre presenting its ordinary physical characters, is excessively rare on the cervix uteri.

2. Ulcerations presenting the characters of the inflammatory ulceration are, on the contrary, exceedingly common in patients labouring under blennorrhagia, or primary, secondary, or tertiary syphilis.

3. Some few of these ulcerations may be primary or secondary, but the very great majority are merely inflammatory.

A short account of cancerous ulceration of the cervix precedes the consideration of the treatment of superficial inflammation and ulceration of the uterine neck.

If inflammation of the mucous membrane of the cervix is ascertained to exist alone, not having passed on to ulceration, the nitrate of silver is found to exercise a most beneficial effect in curing it, just as it does in other mucous membranes, as the conjunctiva, urethra, &c. when similarly affected. The solid caustic may be lightly drawn over the surface, and emollient or slightly astringent injections of lead, zinc, &c. may be used three or four times a-day. The patient must be kept strictly at rest, sexual congress must be forbidden, and the general health attended to. It may be necessary to re-apply the nitrate of silver two or three times, especially if the injections have been negligently or imperfectly used. Dr. B. speaks of the unsatisfactory way in which these local applications are generally made use of, and our own experience most abundantly confirms it. We have long since ceased to depend on vaginal injections as a means of relieving affections of the cervix, or rather we should say, we reserve our doubts of their ever reaching this part. Taking the large run of cases in which they are employed, there are very few that are materially relieved by them, and that too because our directions are not properly carried out. There are some cases which would get well without the use of the nitrate of silver, but the employment of agents locally applied, is not only serviceable from the good they do, but also that the upper part of the vagina is more readily cleansed of any morbid secretion which collects there, and which Dr. Bennet thinks perpetuates inflammation and leads directly to ulceration.

When inflammation of the cervix has run into ulceration, the treatment consists of superficial cauterization with emollient and astringent injections, rest, both of the organ and of the system, and attention to the general health. Dr. B. speaks in the most unqualified terms of the value of cauterization.

"The progress of the inflammation and ulceration is, generally speaking, at once arrested by the cauterization. The congestion and redness of the cervix diminish visibly, the granulations become smaller and healthier, and the purulent secretion assumes the character of laudable pus, if it has not presented it before. When the cauterization is suspended, the ulceration will, however, often remain stationary, even if other measures (injections, &c.) are resorted to; and, if left entirely to itself, it is nearly certain to relapse, however advanced the healing process may be."

The two principal caustics are nitrate of silver and the acid nitrate of mercury. The last, which is used extensively in France, "is a powerful caustic, giving rise to a white eschar, which does not fall for five or six days. When the inflammation is intense, the ulceration large, and the granulations redundant or unhealthy, it exercises a very prompt and beneficial influence, generally cleansing and modifying the sore in one application." Besides these two caustics, Dr. B. mentions the hydrate of potash and the actual cautery. The former is used when neither the nitrate of silver or the acid nitrate of mercury are sufficient for the purpose. It may be used in the same way as the solid nitrate of silver, and afterwards a little water should be injected, or a ball of lint placed against the cervix to defend the adjacent structures. M. Jobert cauterizes nearly all severe uterine ulcerations with the actual cautery, which is scarcely felt, and is free from danger.

In the treatment of that class of cases, where, in addition to ulceration, the central tissues of the cervix have become hardened and hypertrophied, Dr. B. makes the important remark, that, although the former may be cured by the means already indicated, yet, that the presence of the latter state perpetuates the mechanical symptoms, and frequently brings back the ulceration. He finds fault, with great justice, we think, with the

French surgeons for this partial curative treatment, and he lays it down as a rule that, "*whenever* general induration exists, it must from the first be taken seriously into account in the treatment." The management consists in strictly enforcing complete rest, the use of tepid or cold hip-baths and cauterization of the cervix, by the acid nitrate of mercury or caustic potash. These measures should be used for two or three weeks, and if not successful, then leeching the cervix should be had recourse to, which, as might be anticipated, is a useful agent in resolving deep-seated inflammation. While this local treatment is being adopted, attention must be paid to the general health, and any attempt to relieve the partial prolapse of the uterus by pessaries be abandoned.

In order to modify effectually an engorged cervix which has resisted all other modes of treatment, the part should be cauterized deeply, either with the Vienna Paste or the actual cautery. A deep eschar is formed by these means, and when it separates, there is a healthy surface, and a great diminution in the size of the cervix. Dr. B. speaks very positively of the value of this plan of treatment; he gives directions about the mode of applying these caustics, and his experience at La Pitié convinces him that there is but little danger attending their use. M. Gendrin has met with a few cases of intense metritis following the cauterization of the cervix; but, although Dr. B. for three years was in the habit of thus treating two or three cases a week, he never saw any serious result from it. Slight hæmorrhage sometimes follows the separation of the slough.

We confess that we are more disposed to differ with Dr. Bennet on the treatment of these indurations of the cervix than with any other part of his book. The directions for the management of ulcerations in general, are very judicious, and by far the best that can be adopted. We have been in the habit for some time past of treating these cases much in the same way as Dr. B. recommends, and we have every reason to be satisfied with its result. We can, moreover, confidently speak of the perfect fidelity of his descriptions, and we do not think he has left much to be added to the subject. With our author, we think the nitrate of silver by far the most useful application to the ulcerated surface, and its effect in relieving the attendant leucorrhœa is in general speedy and successful. Indeed, we have on several occasions seen symptoms of uterine congestion, with some fever, headache and hurried respiration, succeed its use, from the sudden suppression of the flux, and we think it only prudent to take this contingency into account. Warm fomentations, saline aperients, with antimonial wine and rest, will generally relieve these symptoms. We have found a solution of sulphate of copper, or the direct application of the crystal, useful when the granulations are pale and flabby, or in the aphthous ulceration of the cervix. We cordially concur with our author on the great importance of getting rid of the chronic induration which may thicken the cervix after the ulceration is cured; but we really doubt the propriety of forming deep eschars on the cervix for this purpose. We cannot doubt, after Dr. B's testimony, that they are equal to the resolution of the induration, but surely it is no trifling matter to implant a cicatrix-tissue in this part of the womb. It is to be remembered that, although sterility may have existed for a long time in these cases, yet that the cure of the ulceration is frequently followed by pregnancy. And we are quite sure that Dr. B. will agree with us, that a cervix made rigid by a cicatrix is one of the most trying complications in midwifery. It is just the class of case in which the dilating process is sometimes prevented, and nothing is left but either to divide the unyielding tissue, or run the risk of the lower portion of the womb being separated and cast off. With this fear before us, we confess we have not ventured in these cases to adopt the measures advocated by our author. We rely on leeching or scarifying the cervix, and we then attempt, by the use of iodine or mercury, to resolve the induration. Both these remedies may be locally applied as suppositories introduced at night as high as the cervix, and there left to melt, and so keep the part fully under their influence. The internal exhibition of iodide of potassium is also useful, and although we must own that we have frequently found these indurations very difficult and slow of cure, yet we are able in general to prevent the re-appearance of ulceration, and we think our plan of treatment safer than that of our author and the French surgeons. M. Colombat recommends the use of irrigations or prolonged injections, as well as douches, as powerful means for resolving hard and indolent engorgements, and he describes an apparatus for the purpose.

Our space will not allow us to enter much more fully into the remaining chapters of Colombat's work.

In the treatment of cancer, M. Colombat enumerates the endless variety of remedies—frequently of opposite qualities—which have, at different times and by different persons, been supposed equal to the cure of the complaint. In the surgical treatment of it, he discusses the use of cauterization, of which he says, "that, although it is often efficacious in cases of superficial ulceration, it is always useless and even injurious in extensive ulceration and deep scirrhous degeneration. The re-section of the diseased parts is then the only resource which offers any chance of success." There is a long chapter on amputation of the neck of the uterus, in which the various modes of operating are described. These are introductory to a method pursued by the author, in which we have a new and very complex-looking instrument, which, so far as we can make it out, is much better adapted for a picture, than for the purpose for which it is designed. M. Colombat does not define the class of cases in which this operation ought to be performed. He surely does not mean it to be employed in the last stages of cancer, and yet we should almost gather this from the little that he says on this important practical point.

On the subject of Physometra, we have a strange contrast in the very unpractical text of Colombat, and some very sensible remarks of Dr. Meigs. The latter is a disbeliever in the notion of gas being secreted by, and retained within, the cavity of the uterus; and he explains the supposed cases of physometra in the unimpregnated female, by the lax and flaccid vagina drawing in air when the female lies down, and expelling it again when the hypogastrium is pressed upon, or the patient sneezes or coughs. The same phenomenon constantly occurs, as is well known, after labor.

Colombat describes only two varieties of Polypus—the cellulo-vascular or soft polypus, and the fibrous or hard polypus, of which latter there are two varieties, the pediculated and the sessile. The chapter is a confused one. Dr. Meigs quotes an interesting case from the American Journal of Medical Sciences, related by Dr. John Davis of Smithville, South Carolina, in which a large polypus was spontaneously expelled two days after delivery without hæmorrhage, the polypus being full of large veins and arteries. In the treatment of polypi by ligature, Dessault's instruments and mode of operating are described at length, and, as usual, we have some three or four of M. Colombat's own instruments figured in the act of doing all that could be desired. Strangely enough there is an omission of any mention of Gooch's canula, or of his admirable essay on this subject, and yet, we are quite sure that, for sound discriminating practical knowledge, Gooch's essay is immeasurably superior to Colombat's—just as his simple instrument appears to us to be far better adapted to the removal of polypi than the complicated contrivance of M. Colombat.

In speaking of varices of the vulva as complications of pregnancy, Dr. Meigs tells us that he has had occasion to contend with dangerous crural phlebitis coming on after labor, and clearly taking its rise from the diseased and distended veins of the leg. He lost a case of this kind, in which pyogenic fever, the result of phlebitis, destroyed the patient.

One of the concluding chapters of the work is on Puerperal Fever; and Dr. Meigs supplies a note on the subject, which, for its length and completeness, not only supersedes Colombat's article, but forms a separate thesis on this affection. Dr. Meigs' great object is to inculcate the paramount importance of viewing the disease as essentially inflammatory, and to trust for its cure to bleeding. He is a warm advocate for the views of its nature and relief, which were first promulgated by Dr. Gordon of Aberdeen. Dr. Meigs does not admit of compromise. He will not allow of puerperal poison, or of any thing but the local origin of the disease. "Dr. Collins," he says, "evidently entertains the opinion, adverse to my own, that puerperal fever is a something over and above the local disease, and constitutional affection resulting therefrom." In the use of bleeding he is equally positive. There is to be no flinching—nothing feeble or timid in its employment—no dawdling with the malady; but, as a rule, he thinks Gordon's standard of bleeding to 24 ounces within the first 24 hours of the attack, a correct one. Of course, Dr. M. varies the amount of depletion to the peculiar circumstances of the patient; but every one understands the unsparing, vigorous use of the lancet, which was formerly adopted in these cases. Dr. M. has for years been in the habit, both in private and hospital practice, of pursuing this mode of treatment; and "I have the satisfaction to say," he adds, "that my just expectations of success, founded on the doctrines of Gordon, have rarely been disappointed."

We are perfectly at variance with Dr. Meigs in our views of the origin and the treatment of this most formidable malady, and our inference is, that Dr. M. has been treating in Philadelphia a class of cases which we rarely meet with in London. We gather this from the success which seems to have attended the large depletion which he has adopted. We do not think that even Dr. M. could carry out his views in the cases we are in the habit of meeting in this Metropolis. So deep is the vital depression from the onset of these attacks, that it appears to us quite out of the question to venture on large general bleeding. We have seen this practice adopted on the same theoretical grounds that appear to have guided Dr. M., but it has invariably been abandoned.

We take our leave of Dr. Meigs, hoping that he will not reject our hint, to write a treatise of his own on the diseases of women.

